# Hip-fracture osteosynthesis training: exploring learning curves and setting proficiency standards

**DOI:** 10.1080/17453674.2019.1607111

**Published:** 2019-04-24

**Authors:** Amandus Gustafsson, Poul Pedersen, Troels Boldt Rømer, Bjarke Viberg, Henrik Palm, Lars Konge

**Affiliations:** a Copenhagen Academy for Medical Education and Simulation;; b Orthopedic Department, Slagelse Hospital, Region Zealand;; c Orthopedic Department, Hvidovre Hospital;; d Orthopedic Department, Kolding Hospital;; e Orthopedic Department, University Hospital Bispebjerg;; f Faculty of Health and Medical Sciences, University of Copenhagen, Denmark

## Abstract

Background and purpose — Orthopedic surgeons must be able to perform internal fixation of proximal femoral fractures early in their career, but inexperienced trainees prolong surgery and cause increased reoperation rates. Simulation-based virtual reality (VR) training has been proposed to overcome the initial steep part of the learning curve but it is unknown how much simulation training is necessary before trainees can progress to supervised surgery on patients. We determined characteristics of learning curves for novices and experts and a pass/fail mastery-learning standard for junior trainees was established.

Methods — 38 first-year residents and 8 consultants specialized in orthopedic trauma surgery performed cannulated screws, Hansson pins, and sliding hip screw on the Swemac TraumaVision VR simulator. A previously validated test was used. The participants repeated the procedures until they reached their learning plateau.

Results — The novices and the experts reached their learning plateau after an average of 169 minutes (95% CI 152–87) and 143 minutes (CI 109–177), respectively. Highest achieved scores were 92% (CI 91–93) for novices and 96% (CI 94–97) for experts. Plateau score, defined as the average of the 4 last scores, was 85% (CI 82–87) and 92% (CI 89–96) for the novices and the experts, respectively.

Interpretation — Training time to reach plateau varied widely and it is paramount that simulation-based training continues to a predefined standard instead of ending after a fixed number of attempts or amount of time. A score of 92% comparable to the experts’ plateau score could be used as a mastery learning pass/fail standard.

The incidence of proximal femoral fractures (PFF) has been estimated at 0.1% in industrial countries (Dorotka et al. 2003). These patients take up 1.5% of total hospital capacity and constitute a large part of procedures undertaken in orthopedic departments (http://statbank.dk. Accessed 2018). Patients with PFF are on average > 80 years old and often have comorbidities (Roche et al. 2005), making a strong need for timely and definitive surgery. Internal fixation of hip fractures is a common procedure that orthopedic surgeons must master early in their career.

Inexperienced trainees can contribute to prolonged length of surgery and higher rate of reoperation (Palm et al. 2007) and training on virtual-reality (VR) simulators has been proposed to reduce the burden of surgeons’ early learning curve on patients (Thomas 2013). Several studies describe different types of hip fracture VR simulators and their metrics’ ability to distinguish between novices and experienced surgeons (Tillander et al. 2004, Blyth et al. 2008, Mabrey et al. 2010, Froelich et al. 2011, Pedersen et al. 2014). However, there is very limited evidence on how to set up structured training programs or on setting credible pass/fail standards using PFF osteosynthesis VR simulators.

Training for a certain amount of time or on a certain number of cases is a poor predictor for proficiency and will inevitably lead to trainees performing on variable levels after training. Hence, there is a move away from time-dependent learning and toward proficiency-based learning within medical education. It is therefore prudent to find a benchmark for proficiency (i.e., a pass/fail standard) in a simulated setting before the trainee performs actual surgery under supervision ­(Stefanidis et al. 2012a, Goldberg et al. 2017). This will optimize resources and patient safety by ensuring that each individual trainee spends exactly the required amount of time training on the VR simulator.

Earlier attempts to establish proficiency-based criteria have applied the test a few times or sometimes only once. Such criteria based on data early in the novices’ learning curves and on experts not familiar with the simulator do not add much to the validity argument (Cook 2015). When attempting to support a mastery learning proficiency-based criterion it is more prudent to address the level where participants perform consistently. This is the final, autonomous stage of learning motor skills that is the trademark of experts (Magill 2010). One way to do this is to explore learning curves. Individual trainees have their own learning curve that is typically negatively accelerated, i.e., performance improves considerably in the initial part before entering the plateau phase where additional improvement requires a lot of training/repetitions (Madsen et al. 2014). Another credible way to determine a performance standard is to assess the performance of experts on the same simulator metrics as the trainees (Dyre et al. 2015, Thinggaard et al. 2016)

We determined the characteristics of learning curves for novices and familiarization curves of experts to establish a credible pass/fail mastery-learning standard for junior trainees.

## Methods

The study was conducted from May 2015 to July 2017 at Copenhagen Academy for Medical Education and Simulation, Copenhagen University Hospital Rigshospitalet (Konge et al. 2015a). All training was done during the simulation center’s opening hours and participation was voluntary.

**Figure 1.**
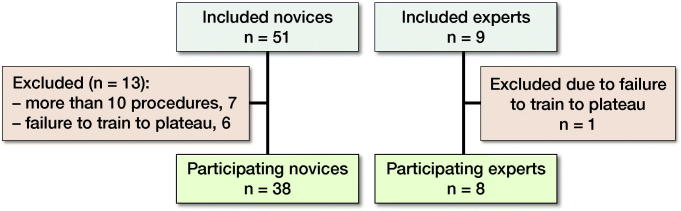
Participants inclusions and exclusions.

51 novices in their 1st year of specialization were included from 7 different departments. 7 novices were excluded as prior to training they had performed more than 10 osteosyntheses of proximal femur fractures under supervision. 6 novices were subsequently excluded as they discontinued training before reaching plateau. 9 experts from 3 different departments who were all consultants with specialization in orthopedic trauma surgery were included. 1 expert was excluded because of failure to test to plateau (Figure 1). We do not know the reasons as to why some participants discontinuing training before plateau in either group.

**Figure 2.**
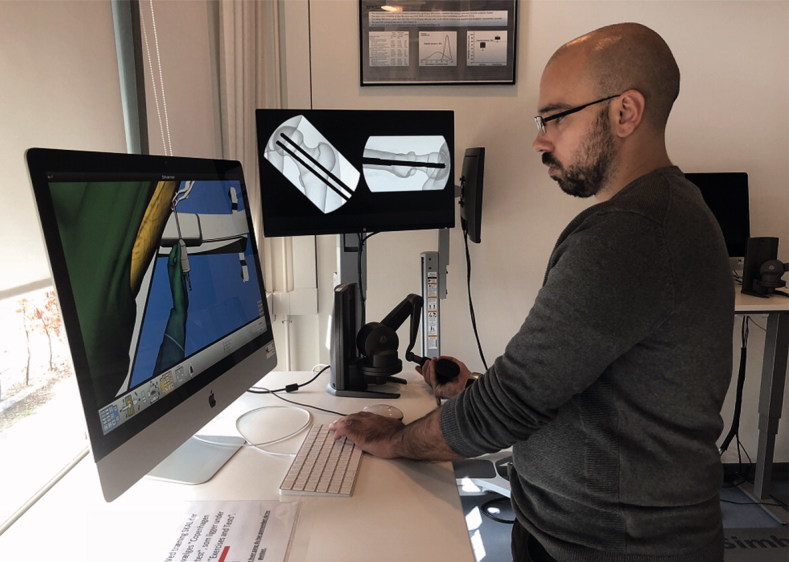
TraumaVision during training.

We used previously validated software on the Swemac TraumaVision simulator (STV; Swemac Osmedic ApS, Nivå, Denmark) to explore learning curves of orthopedic surgeons (Pedersen et al. 2014). The STV simulator consists of a computer with 2 screens and software TraumaVision 5.12. A force feedback device (Phantom Omni; Delft Haptics Lab, Delft, the Netherlands) that mimics the surgery tools and generates haptic feedback is connected to the computer. Either the right or the left hand, according to the preference of the user, can handle the device. The movements are visualized on one of the screens. The fluoroscopy is administered by a foot-paddle and can be displayed in either a standard A-P or lateral view on the other screen (Figure 2). The software contains a variety of orthopedic procedures and the 3 used in our program were cannulated screws, Hansson hook-pins, and a sliding hip screw.

The individual score on each procedure is a percentage of maximum of metrics deemed clinically relevant by the manufacturer and supported by validity evidence (Pedersen et al. 2014). A combined score was produced as a mean of the individual scores. The individual metrics, individual score, and combined score were used to give feedback after completion of the 3 procedures. Only the combined score was used for data analysis as the scores of the individual parameters have insufficient validity (Pedersen et al. 2014).

All participants were naive to the simulations. They were introduced to the simulator and instructed in the correct operation technique for the 3 procedures prior to training. An orthopedic surgeon experienced in hip fracture surgery conducted the introduction and presentation. After completing the introduction, the participants completed a warm-up session containing the 3 procedures. Subsequently the participants trained in 2-hour sessions and received simulator feedback after finishing each round of procedures. The participants were not allowed to train for more than 2 hours per day due to risk of fatigue causing reduced learning (Andersen et al. 2015). A trained simulator assistant oversaw all training and helped the participants interpret the simulator feedback if needed. The training stopped when the participant reached the plateau phase indicated by 3 consecutive attempts without improvement. The plateau criterion was based on a compromise to reduce risk of participant dropout due to prolonged training, while at the same time having an estimated low risk of plateau underestimation. The participants were aware they were training to plateau, but unaware of the plateau criterion.

### Statistics

Levine’s tests were performed and independent samples Student’s t-tests with equal variances assumed/not assumed as appropriate were used to (1) compare the performance of the novice and expert group for variables with normal distribution and (2) to compare continuous data for variables for the novice group. Either Pearson’s chi-square or Fisher’s exact test was used as appropriate to compare categorical data for variables for the novice group. For comparison of performance of the novice and expert group for variables with non-normal distribution, bootstrapped independent samples t-test was used. 95% prediction intervals (PI) for the novices’ training time, best score, and plateau score was calculated using linear regression analysis adjusting for age, sex, dominance, performed procedures, span of orthopedic employment, and previous simulation-based training. The plateau score was defined as the average of the participant’s last 4 scores. The mean plateau score distribution of the 2 groups was plotted using the contrasting groups method (Downing and Yudkowsky 2009). The intersection between the 2 groups was set as a pass/fail standard and the consequences of the pass/fail standard in comparison with the pass/fail mastery criterion were explored. The statistical analysis was performed using SPSS version 22 (IBM Corp, Armonk, NY, USA). Differences in metrics were considered statistically significant when the p-value was < 0.05. 95% confidence intervals (CI) are used.

### Ethics, funding, and potential conflicts of interests

Ethical approval was obtained prior to commencement of the study from the Regional Ethical Committee of the Capital Region in the form of an exempt letter (21/11/2014, No. H-4-2014-FSP). The participants gave informed consent and could opt to drop out at any time. There was no external funding for the study. None of the authors have any competing interests to declare.

## Results

**Figure 3.**
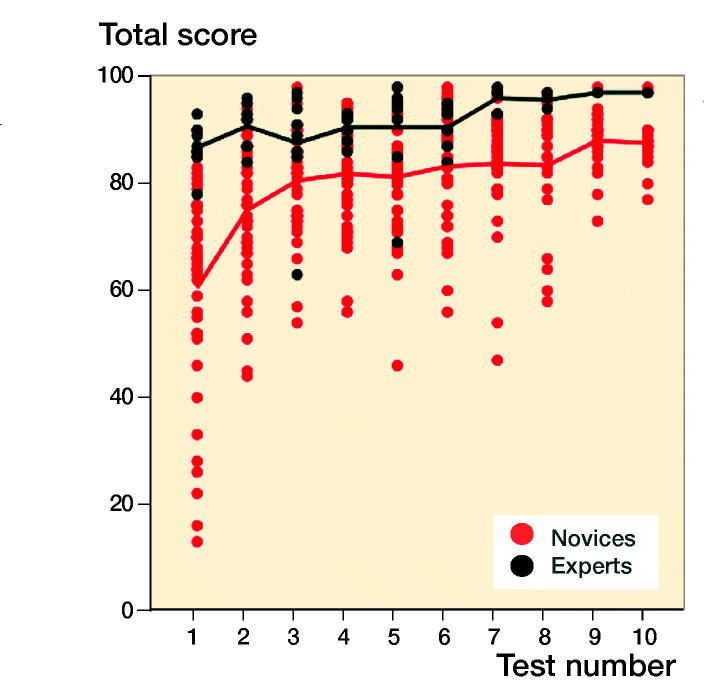
Learning curves for the first 10 attempts of novices and experts.

**Figure 4.**
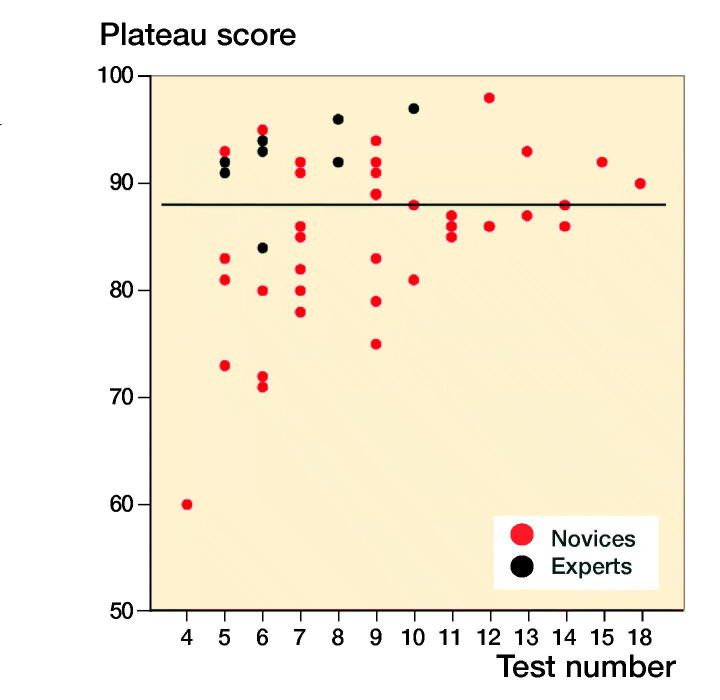
Plateau scores of novices and experts illustrating the large variation in attempts needed to train to plateau. Line at 88% illustrates the consequences of pass/fail standard of contrasting groups method with many novices plateauing well below and above.

**Figure 5.**
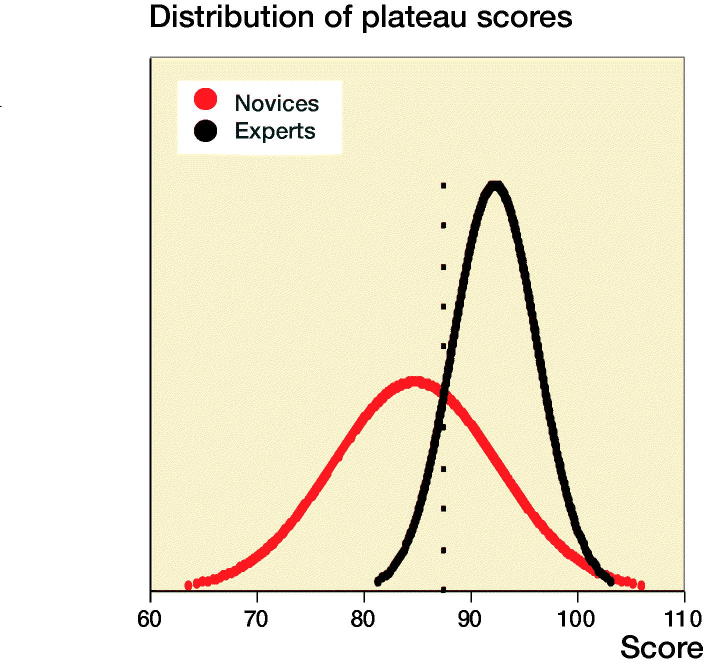
Distribution of plateau scores for novices (red) and experts (black). Using the contrasting groups method, a pass/fail standard for the test can be determined from the intersection of distributions (88%).

For novices the median performed osteosynthesis of proximal femoral fracture was 1 (0–10), employment at an orthopedic department was 7 months (0–22), and age 29 (26–54). The median number of years working full time as orthopedic traumatologist after specialization for the experts was 4 (3–15). The novices had hands-on simulation training for an average of 169 minutes (CI 152–187, PI 162–177) to achieve their learning plateau while experts tested on average for 143 minutes (CI 109–177). The highest achieved scores were 92% (CI 91–93, PI 91–93) and 96% (CI 94–97) for the novices and the experts, respectively (Figure 3). The plateau scores were 85% (CI 82–87, PI 84–86) and 92% (CI 89–96) for the novices and the experts, respectively (Figure 4). When examining demographic and previous experience of the novices who failed to score within 1 standard deviation of the experts’ plateau scores compared with novices with more than 1 SD, no statistically significant difference with regard to age (p = 0.1), sex (p = 1.0), dominance (p = 0.7), performed procedures (p = 0.2), span of orthopedic employment (p = 0.7), or previous exposure to simulation-based training (p = 0.5) was found (Table). A pass/fail standard for the plateau score was defined as 88% using a contrasting groups method (Figure 5). A pass/fail mastery criterion was defined as the experts’ mean plateau score of 92%.

## Discussion

This study demonstrates that the time needed to train to a plateau of consistent performance is highly variable (Figure 4). This makes it essential that a simulation-based training program for novices is not based on time spent training or numbers of repetitions as this inherently will create a substantial risk of either premature termination of training or training with little or no improvement for a high proportion of the trainees. Training must be continued until a predefined criterion based on solid evidence is reached. To our knowledge there are no former studies setting credible mastery-learning pass/fail standards that ensure basic proficiency in simulated hip fracture surgery.

We found that experts performed better both measured by highest obtained score and by plateau score. As expected, experts also had a higher score on the 1st attempt and hence their familiarization curves have a high onset and are relatively flat, improving only slightly with increasing number of repetitions. The novices on the other hand produced a steep learning curve followed by a curve comparable to the experts with more slight progression, but well below the expert curve (Figure 3). The ability of the simulation test to discriminate between novices training to their learning plateau and experts testing to a familiarization plateau widely amplify previously established (Pedersen et al. 2014) validity evidence of the test. It was unexpected that the experts spent similar time to the novices to reach plateau. This finding indicates that the experts need quite some time to get accustomed to features of the simulator that do not resemble their daily clinical life. Change of direction is not allowed when the lateral cortex of the femur is penetrated by the K-wire, making it necessary to retract and reintroduce the wire in the correct trajectory. This feature can be advantageous for motor learning of the novice but produced challenges to the experts who were all accustomed to penetrating the lateral cortex at the correct entry point and then changing trajectory as needed. Another challenge was the distance from the tip of the K-wire to the femoral head joint. The simulator parameter has an acceptable distance of 1–3 mm to reduce the risk of K-wire pullout when the cannulated drill is retracted. Many of the experts were used to inserting the K-wire with a larger distance to the joint surface. As the procedure is autonomous for the experts, they exerted some effort to change strategy to comply with the simulation and parameters.

When examining a passing competence criterion, Pedersen et al. (2014) found a pass/fail standard of 58% using a contrasting groups method, but suggested a score of 75%, based on data from a single performance on the simulator. In our study all novices but 1 achieved a maximum score above 75% and all but 4 achieved plateau scores above this level, indicating that a pass/fail standard for proficiency must be higher to exploit the maximum training effect of the VR simulator in a mastery-learning program. The contrasting groups standard-setting method sets a cut-off where the combination of passing novices and failing experts is at its lowest and is a commonly used method to set a standard for a test and a pass/fail standard for proficiency before supervised practice on patients (Jørgensen et al. 2018, Russell et al. 2018). We used the participants’ plateau scores indicating consistent maximal obtainable scores for the participants to calculate the pass/fail score with this method. The method indicated a score of 88%. However, from Figure 3 it is apparent that a sizeable proportion of the novices achieve plateau scores well below 88% after a few (4–7) attempts. Though it cannot be ruled out that this is due to lack of ability, we would argue that it is likely to be an example of arrested development as described by Ericsson (2009). He argues that most learners, after achieving a standard of performance that can be elicited with reduced concentration, no longer attempt further improvement and development will be prematurely arrested. Our novices trained without the motivation to achieve a predetermined pass score and their plateau score might not represent their best obtainable score. When no standard is set the trainees must rely on their own self-assessment that can be poor in skills training. Andersen et al. (2017) studied novices doing VR simulation-based self-directed training in mastoidectomy and found that training was terminated well before a set time limit when additional time would have permitted better performance. Their learning curve plateaued (too) early as seen for a large subgroup in our study. Likewise, Jowett et al. (2007) demonstrated no superior skills retention with further simulation-based training in knot-tying after the trainees had reached self-assessed proficiency and propose the explanation could be arrested development due to lack of intrinsic motivation. Hence, it can be precarious to base proficiency on data from self-assessed training of novices.

Yudkowsky et al. (2015) argued that standards derived from other novices have no place in a proficiency-based curriculum or mastery setting and emphasize overlearning and automaticity as conveyors of long-term retention. In accordance, Stefanidis et al. (2012b) have shown that novices training above and beyond standard proficiency levels set by expert performance have superior skill acquisition and transfer compared with training to proficiency alone. Madsen et al. (2014) explored consequences of performance standards based on the contrasting groups method and experts’ levels on a transvaginal ultrasound simulator test and found that the novices were readily able to train to expert-level scores and that using lower standards should not be recommended. In our opinion the raison d’être for simulation-based training is promoting mastery learning through deliberate practice and we therefore suggest a pass/fail plateau score of 92%, comparable to the average for experts before trainees progress to supervised practice in the clinical setting.

The simulator allows for anatomical variation with 4 skeletons integrated in the software and allows for training on suboptimally reduced fractures. A limitation to our test is that it was chosen to base the training on exclusively anatomical reduced fractures on one skeleton’s left side and hence without variation of anatomy. This could lead to higher scores and faster plateauing for the novices compared with the experts, as it can be argued that it is technically more challenging to place the implants optimally in less than perfectly reduced fractures with anatomical variation. This supports the validity argument of the test, but the competency criterion proposed cannot necessarily be transferred to a training setup where variation is introduced. This can be desirable, as variation during training has been shown to enhance learning outcomes in simulation-based training (Zendejas et al. 2013). Another important implication to consider when interpreting consequences of the competency criterion is the inherent limitation of data based solely on simulation. Even though studies on other simulators and settings have explored consequences of the mastery criterion as a pass/fail standard and found it feasible, it is not axiomatically so in the present setting. The expert group sample is small and may represent a level of skill on the simulator that is unobtainable for some novices regardless of interventions to improve learning during training. The impact can be undesirable training that has no improved effect in the clinical setting—the intrinsic factor of application of simulation-based training. To that end, the present mastery criterion is suggestive as transference of skills to the clinical setting and optimal training for this effect is still unknown.

Skills obtained from VR training have been shown to be transferable to the clinical setting within many medical specialties (Konge et al. 2015b, Tolsgaard et al. 2017, Thomsen et al. 2017). However, to our knowledge, only Howells et al. (2008), on a bench-top arthroscopic knee model, and Cannon et al. (2014) on VR knee arthroscopy simulator have shown transfer of skills to the operating theatre within the field of orthopedic surgery. It is essential that future research within simulation-based training focus on transfer of skills to the clinical setting to optimize training and gauge the consequences of competence criteria of simulation-based training.

In summary, this study found that the time training to plateau displayed a high degree of variability. Experts achieved higher scores through all phases of the learning curve compared with novices, supporting enhanced validity evidence of the test, and we suggest a credible pass/fail score of 92% as an average of 4 consecutive attempts before novices proceed to supervised practice on patients. It is important in future research to address the transferability of skills obtained from this simulator to clinical practice and the consequences of passing criterions.

AG: study design, data collection, statistical analysis, data analysis and interpretation, writing of the manuscript. PP: study design and revision of the manuscript. TBR: data collection and revision of the manuscript. BV: study design and revision of the manuscript. HP: study design and revision of the manuscript. LK: study design, statistical analysis, data analysis and interpretation, revision of the manuscript.

*Acta* thanks Katre Maasalu and Sari Ponzer for help with peer review of this study.

**Table:** Demographics and previous experience for the novices grouped by score within and more than 1 SD of experts’ plateau score

	n	Mean age (CI)	Male/ female	Dominant hand (R/L)	Mean OE (CI)	Mean SP (CI)	Previous simulation (yes/no)
Within 1 SD	13	27.9 (27.2–28.6)	9/4	10/3	6.8 (4.2–9.4)	2.3 (0.9–3.8)	7/6
More than 1 SD	25	30.4 (28.2–32.6)	16/9	21/4	5.5 (6.7–11.1)	2.7 (1.4–4.0)	10/15
p-value		0.1	1.0	0.7	0.7	0.2	0.5

R/L = right/left. OE = orthopedic employment. SP = supervised procedures. SD = standard deviation.
